# Differences in cognitive aging: typology based on a community structure detection approach

**DOI:** 10.3389/fnagi.2015.00035

**Published:** 2015-03-19

**Authors:** Emi Saliasi, Linda Geerligs, Jelle R. Dalenberg, Monicque M. Lorist, Natasha M. Maurits

**Affiliations:** ^1^Department of Neurology, University Medical Center Groningen, University of GroningenGroningen, Netherlands; ^2^NeuroImaging Center, University Medical Center Groningen, University of GroningenGroningen, Netherlands; ^3^Department of Experimental Psychology, University of GroningenGroningen, Netherlands

**Keywords:** aging, cluster, community structure, neuropsychological test, intelligence

## Abstract

The current study investigated the extent and patterns of cognitive variability in younger and older adults. An important novelty of this study is the use of graph-based community structure detection analysis to map performance in a mixed population of 79 young and 76 older adults, without separating the age groups a-priori. We identified six subgroups, with distinct patterns of neuropsychological performance. The stability of the identified subgroups was confirmed by employing a cross-validation support vector machine based analysis. The majority of these subgroups comprised either young or older adults, confirming the expected role of aging in cognitive performance. In addition, we identified a subgroup of young and older adults who performed at a similar cognitive level of overall good cognitive performance with slightly decreased processing speed. This result showed that older age is not necessarily associated with general lower cognitive performance and that being young is not necessarily associated with superior cognitive performance. Moreover, cognitively better performing elderly had a significantly higher level of education attainment and higher crystallized intelligence than the other elderly, which suggests that older adults with higher cognitive reserve may be able to cope better with age-related neurobiological change.

## Introduction

Healthy aging has generally been associated with a decline in cognitive task performance (Salthouse, 1996; Hedden and Gabrieli, 2004; Park and Reuter-Lorenz, 2009). However, the trajectory and degree of age-related cognitive change vary considerably across individuals. The interindividual variability in cognitive performance (on a single task and occasion, referred to as “diversity” (Nelson and Dannefer, 1992)), but also intraindividual variability in cognitive performance (on multiple tasks, referred to as “dispersion” or on a single task on multiple occasions, referred to as “inconsistency”) increase with age (Hultsch et al., 2002; Wilson et al., 2002). Only few studies have investigated variability of cognitive performance across tasks and across elderly, combining diversity and dispersion. In one of these studies, Costa et al. (2013) divided older adults (50–89 years) into three groups of generally stronger, average and weaker cognitive performers (Costa et al., 2013). The results showed that these groups had stable performance across the cognitive dimensions that were taken into account. In contrast, Gunstad et al. (2006), found distinct performance profiles across cognitive domains in the groups they distinguished (Gunstad et al., 2006). In their subsample of 84 older adults (50–82 years), one group showed impaired executive functioning, a second group performed poorly on tasks measuring processing speed while a third group showed a more general decrease in overall cognitive performance. A similar distinction in performance profiles has been reported in other studies (Ritchie et al., 1996; Maxson et al., 1997; Ylikoski et al., 1999; Foss et al., 2009; Costa et al., 2013) that specifically assessed cognitive typology in elderly.

Most previous studies examining variability in cognitive aging, have focused on older adults only (Ritchie et al., 1996; Maxson et al., 1997; Ylikoski et al., 1999; Foss et al., 2009; Costa et al., 2013) or have used an a-priori division of their research population in different age categories (Gunstad et al., 2006). It is important to note that variability in cognitive performance among the elderly might be explained by factors that are already present at younger ages. For example, genetic modulation of cognition (Lindenberger et al., 2008; Nagel et al., 2008) or of dopamine receptors (Bäckman et al., 2011; MacDonald et al., 2012) may explain cognitive performance variability, also in younger adults. However, the influence of these factors might change with age. Neurobiological differences (Myerson et al., 1990) and decline in the efficiency of executive control (West et al., 2002), for example, are thought to increase cognitive performance variability with advancing age. In addition to these internal factors, environmental factors such as socioeconomic status, education level and intelligence quotient (IQ) might influence variability in cognitive performance (Stern, 2002; Foss et al., 2009; Tucker-Drob et al., 2009). These external factors are thought to allow some to cope better with the neural and cognitive decline in the aging brain, than others (cognitive reserve theory (Stern, 2002, 2009; Steffener and Stern, 2012)).

The current study aimed at extending our knowledge on the diversity and dispersion in cognitive performance. Profiles of cognitive performance observed in the elderly may not be just related to age. Therefore, we here derive a typology of cognitive aging in a mixed group of young and older adults. Thus, we do not assume a-priori that younger and older adults have different cognitive profiles. In addition, in this study we focus on compound neuropsychological test results, which are frequently used in clinical settings, to evaluate functioning across a variety of cognitive domains. Our results, therefore, might have a direct clinical impact allowing a better dissociation of patterns of cognitive decline resulting from either healthy aging or specific disease, which is especially useful in clinical decision-making (Geldmacher et al., 2012).

Various clustering techniques can be applied to identify cognitive typologies based on neuropsychological test results. In general, these techniques can be divided in three well-known classes: (1) hierarchical methods, that cluster data points on the basis of distance connectivity (e.g., Ward’s method or linkage hierarchical methods (Maxson et al., 1997; Passarino et al., 2007)); (2) centroid methods that represent clusters by a central data point, that may not be part of the dataset (e.g., K-means clustering (Maxson et al., 1997; Ylikoski et al., 1999; Newman, 2004)); and (3) distribution-based methods that define clusters as data points most likely belonging to the same distribution (e.g., Bayesian latent class analysis (Costa et al., 2013)). Although all clustering methods have their advantages and disadvantages, we have favored the application of the first mentioned hierarchical method in our analyses since it dovetails nicely with basic concepts in graph theory (i.e., distance connectivity has a natural counterpart in graphs) and can therefore profit from recent advances in graph theoretical clustering approaches.

Graphs, as used in graph theory, are sets of nodes or vertices connected by lines or edges. Data points, making up the nodes in a graph, can have high similarity (i.e., resulting in connected nodes) or low similarity (i.e., resulting in unconnected nodes). Based on the connectivity pattern between data points, a graph can be constructed for any data set. To cluster the data points (i.e., nodes in the graph), community detection can be applied to the graph. Community detection identifies groups of nodes (i.e., clusters) that are more densely interconnected than they are connected with the rest of the nodes. One of the most widely used methods for community detection is modularity maximization (Newman, 2004). Modularity is a graph-theoretic measure that quantifies the quality of a particular division of a network into communities. However, modularity maximization is a very computationally intensive process when an exhaustive search is used. One of the most efficient alternative methods today is the method proposed by Newman (2006), which reformulates modularity in terms of the spectral properties of a network and can be seen as a hierarchical clustering method. Since its inception, this method has been amply used, for example, to identify cognitive typologies in typically developing youth and in children with ADHD (Fair et al., 2012). In the current study, we applied this method to identify cognitive typologies in a mixed group of young and older adults.

In our application, the graph consists of nodes reflecting participants and connections between them index the similarity of cognitive test performance between the participants. Because we know that cognitive aging is a highly variable process and that cognitive performance of some elderly is on the same level as that of their younger counterparts, we hypothesize that the chosen method applied to a group consisting of both young and older adults will identify at least one subgroup of cognitively similarly performing younger and older participants. In addition, we also expect that several older adults will be separated from the young adults, in line with theories of generally lower cognitive performance in older age. In line with the predictions of the cognitive reserve theory (Stern, 2002, 2009; Steffener and Stern, 2012), we expect that elderly with a higher level of education attainment and IQ will show an overall higher level of cognitive performance. Hence, we additionally investigated whether broad measures of functioning (education attainment and estimates of intelligence) that were not included in the determination of cognitive typologies, were related to group membership of individual participants.

## Materials and Methods

### Participants

Neuropsychological data from 158 healthy adults were evaluated for this study. Eighty participants were younger adults (mean age 20.2 years; range 18–26 years; 41 males) and 78 participants were older adults (mean age 65.3 years; range 59–74 years; 38 males). Participants were recruited through advertisements in local newspapers. All participants were right handed and had normal or corrected to normal visual acuity. Exclusion criteria were a history of neurological, psychiatric or vascular disease and use of any psychotropic medication. To verify normal overall cognitive functioning, the MMSE (Folstein et al., 1975) and the HADS (Zigmond and Snaith, 1983) were used. Only participants who scored above 26 on the MMSE and below 16 on each of the subscales of the HADS were included. One younger and one older participant did not complete all neuropsychological tests and were excluded from further analysis. In addition, one older participant was excluded due to brain abnormalities discovered in the anatomical scan collected for other purposes. The local ethics committee approved the current study. All participants gave written informed consent.

### Neuropsychological Testing

Participants were tested on a clinical neuropsychological test battery that encompassed tests for different aspects of cognitive functioning, such as processing speed, executive functioning, verbal fluency, working memory span, recall and recognition, and response speed.

To assess cognitive *processing speed*, the trail making tests A and B were used. The trail making B test has also been associated with *executive functioning* (Lezak et al., 2004). Participants were instructed to execute the tasks as quickly as possible. Both tests were practiced prior to the assessment. These tests were scored by the time taken to complete the test, including the time it took to correct the (possible) errors made. *Verbal fluency* was assessed by four subtests. For two of these subtests (phonemic fluency), participants had to generate as many meaningful words as possible beginning with (1) the letter “S” and (2) the letter “F” in 60 s. For the remaining subtests (semantic fluency), they were instructed to generate as many (1) professions and (2) animals as possible within 60 s. The order of the phonemic and semantic subtests was semi-randomized between participants. The score on each of the fluency tests was the number of correct words. The digit-span tests forward and backward were used to assess *working memory span*. The score on each of the tests was the maximum number of correctly recited digits. The 15 words test was used to assess immediate and delayed *recall* as well as* recognition*. The immediate recall consisted of five blocks of trials: in each block the participants were required to recall as many words as possible, immediately after they were presented to them. The delayed recall and the recognition subtests consisted of one block each. For each of the three subtests, the score was the number of correctly remembered words. Finally, *response speed* was assessed by means of a simple reaction time test, in which participants were required to press a response button as quickly as possible whenever a red dot appeared on the screen. The red dot remained on screen for 300 ms and intertrial intervals (ITI) varied randomly between 2000 and 6000 ms. Response speed was scored as the median response time (RT) for correct button presses.

In addition to the above-mentioned tests that measure specific cognitive functions, tests assessing broader measures of cognitive functioning were implemented. The digit symbol coding test employs nine pairs of numbers and abstract symbols. First, participants memorized the number-symbol pairing and practiced briefly. Subsequently, participants were asked to write down the corresponding symbols under a sequence of numbers, as quickly as possible within 120 s. An estimation of crystallized intelligence was obtained through the Dutch Adult Reading test, which is the Dutch version of the National Adult Reading Test (NART; Schmand et al., 1992) and requires participants to read aloud a list of words, with irregular pronunciation. An estimate of fluid intelligence was obtained from the WAIS-matrix reasoning test in which participants are presented with 26 incomplete patterns (or matrices) and are required to select the response that completes each pattern (Uterwijk, 2001). To estimate education attainment four levels were distinguished: (1) lower education; (2) lower-technical and vocational training and lower general secondary education or preparatory middle-level applied education; (3) vocational training and higher general continued education or preparatory scholarly education; and (4) higher professional education or university level.

To investigate whether the level of education attainment, estimates of fluid and crystallized intelligence, as well as scores on the digit-symbol coding test were related to group membership of individual participants we tested subgroup differences on these variables using univariate ANOVA (significance level *α* = 0.05). To investigate subgroup differences in education level, chi square (*X*^2^) testing was performed.

### Variable Selection

To identify diversity and dispersion in performance on the neuropsychological tests the following 8 compound scores were taken into account for further analysis: (1) phonemic fluency (mean score on the subtests “S” and “F”); (2) semantic fluency (mean score on the subtests “professions” and “animals”); (3) working memory span (mean score of the digit-span forward and backward tests); (4) trail making A score (time to complete the trail making A test); (5) trail making B/A score (time to complete the trail making B divided by the time to complete trail making A test); (6) immediate recall (sum of recalled items in the 5 sessions of the immediate recall subtest); (7) delayed recall score; and (8) response speed score. The recognition score for the 15 words test was excluded for further analysis; due to the lack of variability among participants inclusion of this test would not add relevant information to the identification of cognitive profiles.

### Subgroup Identification: Community Structure Detection

All compound scores were transformed to *z*-scores. Subsequently, some scores were multiplied by −1 to ensure that a higher score was equivalent to better performance. To be able to apply community detection, we first determined the graph describing the relation between participants (i.e., nodes) based on their cognitive test performance. Connectivity between nodes (i.e., similarity in cognitive test performance between participants), was based on the intraclass correlation coefficient ICC (A,1) (McGraw and Wong, 1996) calculated between all pairs of participants across test scores. Subsequently, a square symmetric ICC matrix (155 × 155) was constructed, containing the ICC values for each pair of participants. The ICC matrix was thresholded such that every participant was connected to at least one other participant (the graph was “strongly connected” that is “reachability” was 1; see Fair et al., 2012), resulting in a threshold of 0.3870. Because the threshold could have an impact on the detected communities (Palla et al., 2005), the robustness of the detected communities was further investigated for different (lower) thresholds (ICC = 0.3, ICC = 0.2, ICC = 0.1). For higher thresholds, the reachability of the resulting graph is expected to be lower. We found that the number and size of identified communities was independent of the chosen threshold.

To identify the communities (i.e., clusters or subgroups) in our graph, the modularity (Q) maximization approach of Newman (2006) was used. Newman’s algorithm aims at identifying communities in a network, which share fewer edges between each other than would be expected in a network with an equivalent degree of distribution, in which edges are placed at random. Q quantifies the difference between the actual connections in the network and the expected connections in the equivalent random network; a positive Q thus indicates that the number of edges within communities is higher than expected in the equivalent random graph.

### Cluster Stability and Validation

Participants were assigned to separate clusters using community detection analysis. This method does not provide information on how stable the identified clusters are and how well an individual can be captured within one of the existing clusters or classified as member of an existing cluster. To determine cluster stability, and to indicate how well new individuals can be classified as member of the existing clusters, we used a support vector machine (SVM) with a radial basis function (RBF) kernel, provided in the package LIBSVM (Chih-Chung and Chih-Jen, 2011).^1^ Typically, an SVM is trained on a number of entities (in this case participants) described by a set of defining variables (in this case compound neuropsychological test scores) and their associated class label of each entity (in this case the label of the cluster the participant was assigned to by community detection analysis). Within the training part, the SVM associates patterns among the variables with the class labels. The result is captured in a model, which is able to classify new entities to the existing class labels (see Burges, 1998 for an extensive overview of SVM). The performance of the SVM prediction is often expressed in terms of sensitivity and specificity. Sensitivity refers to how well a class can be predicted and is calculated by the number of true positives in the prediction divided by the total number of true class members. The specificity refers to how specific a group was in the prediction results and is calculated by the number of true negatives in the prediction divided by the total number of other class members.

To assess the SVM prediction performance resulting in clusters that were produced using community detection analysis, we performed two cross-validation procedures; leave one out cross validation (LOOCV) and dataset partitioning (Arlot and Celisse, 2010). In LOOCV, data from one participant is used as testing dataset, while data from the remaining participants forms the training data. This procedure is repeated until data from each participant has been used once for testing purposes. Because LOOCV can give an optimistic result, we also determined the cross-validation results of dataset partitioning. In this procedure, every subgroup identified through community detection analysis was divided in two parts of (almost) equal size. One of these parts was used for training while the other part was used for testing purposes. Cross-validation is a nearly unbiased method to assess how results will generalize to independent data sets. This technique is especially useful when data is scarce and further samples are costly to collect.

## Results

### Community Detection

**Figure 1:**
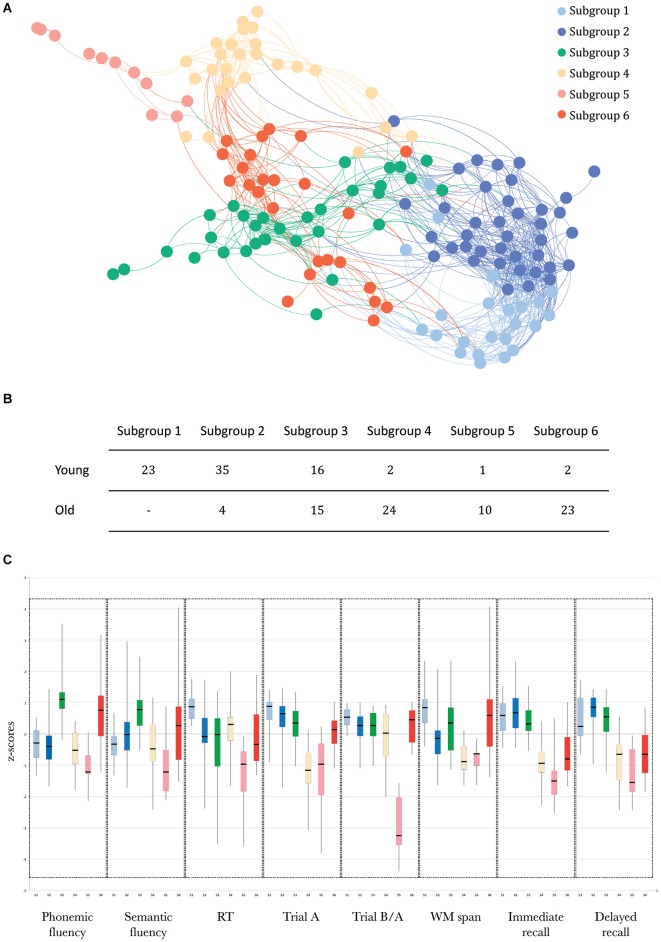
**(A)** Graph representation of the subgroups resulting from community structure identification, using the build-in layout Force-Atlas method in Gephi, version 17 (Bastian et al., 2009). This method employs a spring-directed algorithm that assumes two competing forces, a repulsive force driving all nodes apart and an attractive force (“spring force”) keeping the nodes linked by edges together. The stronger the connection (similarity), the stronger the attractive force and the closer the edges will be in the visualization. **(B)** The number of younger and older adults in each subgroup. **(C)** Boxplots of the scores (median, 1st quartile, 3rd quartile, min and max) for each subgroup for each of the neuropsychological tests, using the same color coding as in panel **(A)**.

Community detection analysis resulted in six separate communities (Figure 1A), at a modularity maximization index (Q) of 0.49. Two of these subgroups consisted mainly of younger adults (Subgroups (S)1 and S2; Figure 1B). One “mixed” subgroup contained comparable numbers of younger and older adults (S3). The remaining three subgroups were dominated by older adults (S4, S5 and S6).

The SVM results, indexing how well participants were assigned to the subgroups identified by community detection analysis, showed that there was a strong distinction between these subgroups (overall classifier accuracy: LOOCV: 83.9% and data partitioning: 74.7%). The sensitivity and specificity of both analyses for each of the subgroups are presented in Table 1.

**Table 1:** **Sensitivity and specificity of the SVM algorithm for each subgroup, for the LOOCV method and the data partitioning method, separately**.

	Subgroup	LOOCV		Data partitioning^1^	
	*Size*	*Sensitivity*	*Specificity*	*Sensitivity*	*Specificity*
**S1**	23	82.6	99.2	83.3	100
**S2**	39	89.7	94.1	65	98.1
**S3**	31	87.1	92.3	93.3	84
**S4**	26	84.6	96.9	84.6	92.8
**S5**	11	81.8	99.3	83.3	100
**S6**	25	72.0	98.4	46.2	96.7

*^1^For the data partitioning procedure, the training dataset consisted of 76 participants: S1(11), S2(19), S3(16), S4(13), S5(5) and S6(12). The testing dataset consisted of 79 participants: S1(12), S2(20), S3(15), S4(13), S5(6) and S6(13)*.

**Figure 2:**
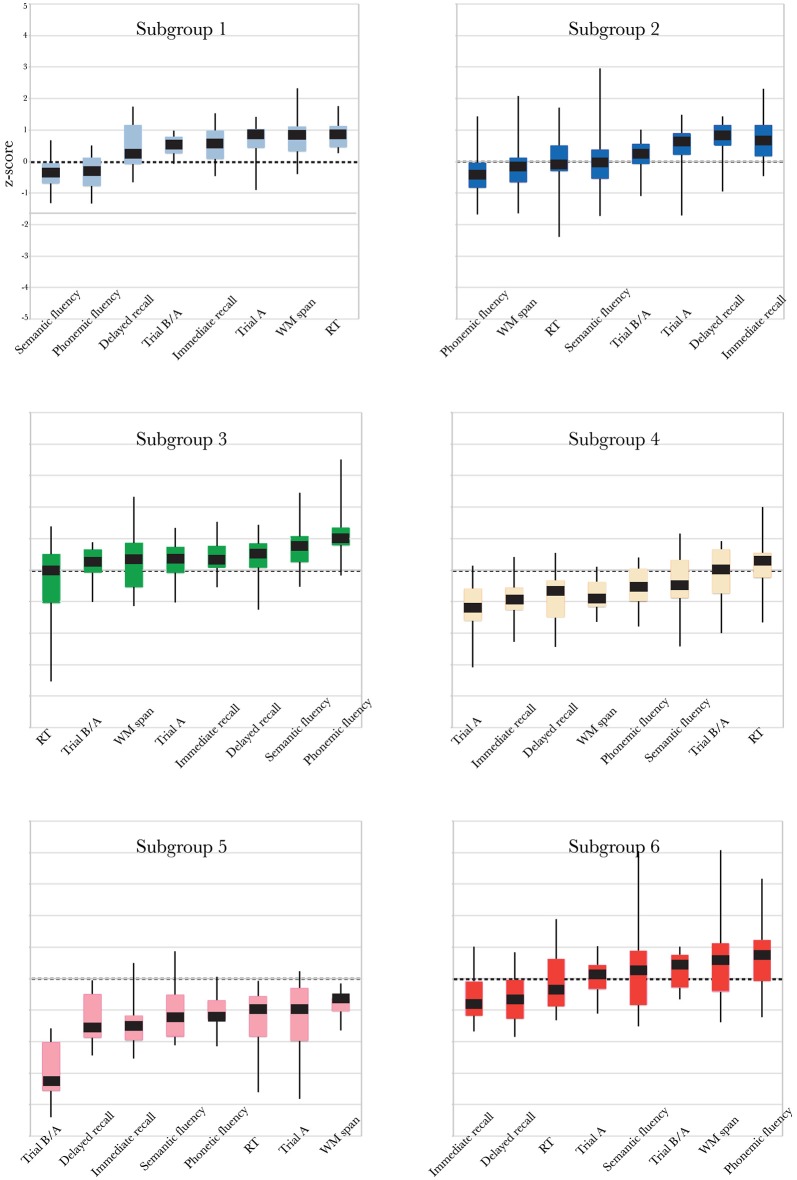
**Boxplots representing compound neuropsychological test scores (median, 1st quartile, 3rd quartile, min and max), separately, for each of the six subgroups**. Note that the order of the tests on the *x*-axis differs between subgroups, because it is ordered by increasing *z*-score. The same color coding is used as in Figures 1A,C.

The profiles of compound neuropsychological test scores are presented in Figure 2, separately, for each of the six subgroups. The cognitive profile of S1 (young, 14.8% of the total group of participants) was characterized by a below average performance on verbal fluency tests and above average compound scores on the remaining neuropsychological tests. Participants in S2 (mostly young, 25.2% of the participants) performed above average on tests related to executive functioning (trail making A and B/A) and memory (immediate and delayed recall), but average or below average on tests related to verbal fluency, working memory span and RT. Participants in S3 (mixed age, 20% of the participants) were generally slower than average (RT), but scored above average on the remaining neuropsychological tests. In contrast, participants in S4 (mostly old, 16.8% of the participants) were faster than average (RT), but had below average compound scores on all remaining tests. The cognitive profile of participants in S5 (mostly old, 7.1% of the participants) was characterized by overall decreased compound scores, particularly on the trail making B/A test that is thought to provide a measure of executive functioning. Participants in S6 (mostly old, 16.1% of the participants) had lower compound scores on tests measuring aspects of memory (delayed and immediate recall) and RT, but average or above average compound scores on the remaining cognitive tests.

### Differences in Demographics and Broad Measures of Cognitive Functioning Between Identified Subgroups

**Figure 3:**
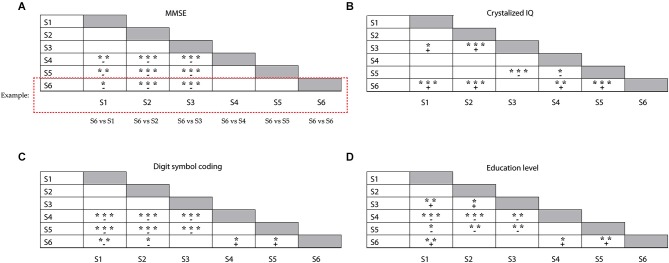
**Illustration of the differences between subgroups, for (A) the MMSE test results, (B) crystallized IQ scores, (C) Symbol coding test scores and (D) education level**. Groups represented in each row were compared with the subgroups represented in each column (e.g., S6 (row) vs. S1 (column), S6 vs. S2 (column); see example given); + reflects a positive mean difference (“row” group larger than “column” group) between the subgroups, – reflects a negative mean difference (“row” group smaller than “column” group) between the subgroups; *p < 0.05; **p < 0.01; ***p < 0.001.

Demographics and broad measures of cognitive functioning (level of education attainment, estimates of fluid and crystallized intelligence, scores on the digit symbol coding test) are presented in Table 2, separately for each subgroup. We analyzed differences in age *distribution* between subgroups, separately for younger and older adults within the groups. Only two subgroups differed with respect to the age of the older participants within the subgroup; older participants in S5 were on average older than older participants in S3 (*F*_(4,71)_ = 3.2; *p* = 0.019). The young adults in each subgroup had comparable age. There were no differences in MMSE scores between the subgroups dominated by younger adults or between those dominated by older adults. However, participants in the younger (S1 and S2) and mixed subgroup (S3) had higher MMSE scores than participants in the older subgroups (S4–S6, *F*_(5,149)_ = 13.8; *p* < 0.005; illustrated in Figure 3A). The six subgroups had similar scores on both the anxiety (*F*_(5,149)_ = 0.84; n.s.) and the depression (*F*_(5,149)_ = 1.7; n.s.) subscales of the HADS test. Participants in mixed subgroup S3 and in the “predominantly” older S6 group, had higher crystallized IQ scores than participants in the two younger subgroups (S1 and S2) and in one older subgroup (S5) (*F*_(5,149)_ = 12.6; *p* < 0.0005; Figure 3B). In general, participants in S5 had lower fluid IQ scores than participants in the other subgroups (*F*_(5,149)_ = 5.1; *p* < 0.0005). Performance on the digit symbol coding test differed between subgroups (*F*_(5,149)_ = 17.7, *p* < 0.0005; Figure 3C). Participants in S1 and S2 had higher scores than all three older groups (S4, S5 and S6). Among these older groups, participants in S6 performed best at the digit symbol coding test. Finally, participants in S6 also attained a higher education level than participants in the other older subgroups S4 ($X_{\left(51\right)}^{2}$ = 8.1, *p* = 0.017) and S5 ($X_{\left(36\right)}^{2}$ = 11.7, *p* = 0.007; see Figure 3D). There were no differences in education level between the two subgroups dominated by younger adults.

**Table 2:** **Demographics and broad measures of functioning for each subgroup**.

	S1	S2	S3	S4	S5	S6
***N***	23	39	31	26	11	25
**Young/Old**	23/-	35/4	16/15	2/24	1/10	2/23
**Male/Female**	13/10	17/22	12/19	15/11	5/6	16/9
**Age (years): young (median(range))**	19(18–23)	20(18–24)	20.5(18–26)	19.5(18–21)	19(19)	21(20–22)
**Age (years): old (median(range))**	-	62.5(59–69)	63(60–70)	65.5(60–74)	68(62–74)	64(60-72)
**MMSE (mean(SD))**	29.4 (0.7)	29.5 (0.6)	29.6 (0.6)	28.4 (1.1)	28.1 (1.2)	28.5 (1.1)
**HADS anxiety (mean(SD))**	3.8 (1.7)	3.2 (2.2)	3.9 (2.8)	3.7 (2.2)	4.6 (2.7)	3.2 (2.9)
**HADS depression (mean(SD))**	1.4 (1.6)	1.8 (1.9)	1.5 (1.6)	2.3 (2.6)	3 (2.9)	1.6 (1.2)
**Crystallized IQ (mean(SD))**	103.7 (5.6)	102.3 (4.9)	111 (8.7)	105.2 (10.4)	96.5 (7.8)	114 (9.3)
**Fluid IQ (mean(SD))**	113 (12.6)	111.2 (10)	111.6 (9.1)	106.5 (8.6)	97.3 (9.3)	111 (9.2)
**Digit symbol coding (mean(SD))**	86 (12.7)	83 (15.1)	79.5 (14)	60.5 (8.3)	56.2 (13.5)	72.6 (13.3)
**Education attainment level (1/2/3/4)**	-/-/18/5	-/-/24/15	-/-/11/20	-/7/6/13	1/3/5/2	-/-/10/15

*S = Subgroup; Education level: 1 = lower education; 2 = lower-technical and vocational training, lower general secondary education or preparatory middle-level applied education; 3 = vocational training, higher general continued education or preparatory scholarly education, 4 = higher professional education or university level*.

## Discussion

In the current study, we investigated variability of cognitive performance across tasks (dispersion) and across individuals (diversity), with the aim to derive cognitive profiles in a population of both young and older adults. Cognitive profiles were identified by community detection analysis (Newman, 2006) on compound scores obtained from a clinically employed neuropsychological test battery. The robustness of the resulting subgroup partition, that is the degree to which new individuals will appropriately be classified as member of an existing subgroup, was confirmed by two SVM-based approaches, LOOCV and cross-validation. A particular strength of the current study is the implementation of the cross-validation SVM approach, which is a nearly unbiased method to assess how results will generalize to independent data sets. This technique was especially useful in the current study in which we investigated cognitive patterns in a relatively small group of participants. We hypothesized that our approach would identify at least one age-mixed subgroup of cognitively similarly performing participants and, in line with theories of general cognitive decline with age, we also expected additional subgroups in which older adults would be separated from the young adults.

Indeed, we identified a mixed subgroup of older and young participants who performed at a similar cognitive level, showing overall good cognitive performance with slightly decreased processing speed. We also found subgroups mainly consisting of younger or older adults. The two “younger” profiles showed overall good cognitive performance, although they both showed relative underperformance in phonemic fluency. The presence of three older subgroups, compared to only two younger subgroups, seems to confirm increased performance variability in the elderly (Hultsch et al., 2002; West et al., 2002). While one of the “older” profiles was characterized by general lower cognitive performance; the other two “older” profiles only showed lower performance in specific cognitive domains in combination with cognitive performance in the range of younger adults on the remaining cognitive tests. All three “older” profiles were characterized by lower cognitive performance on immediate and delayed recall tests, reflecting impaired recollection in the elderly possibly due to age-related frontal lobe dysfunctions (see Yonelinas, 2002 for a review).

Our approach of clustering in a mixed group containing both young and older adults shows that aging is not necessarily associated with poor cognitive performance and simultaneously, that being young is not necessarily associated with superior cognitive performance. Moreover, the results show that the degree of age-related lower cognitive performance seems to vary significantly between individuals. We also found that cognitively better performing participants (older subgroup 6 (S6)) had a significantly higher level of education attainment and higher crystallized intelligence than the participants in the other older subgroups (S4 and S5). Cognitively better performing individuals also showed higher general cognitive competence, reflected in the higher scores on the digit symbol coding test, which draws among others on visuomotor coordination, sustained and selective attention and associative learning (Lezak et al., 2004). Chronological age cannot explain the differences between the older subgroups on these broad measures of cognitive functioning, as the age distribution of the elderly in subgroups 4, 5 and 6 was comparable. Therefore our results seem to suggest that older adults with a higher so-called “cognitive reserve”, as reflected in higher educational attainment and crystallized intelligence level, show a more favorable pattern of cognitive performance. This is in line with previous studies (Ylikoski et al., 1999; Foss et al., 2009) which have suggested that considering the environmental context (e.g., social-economic status, education level) is important in understanding the cognitive trajectory over the adult lifespan (Gribbin et al., 1980; Manton et al., 1986).

The cognitive reserve theory (Stern, 2002, 2009) proposes that higher education level and IQ scores are protective factors that allow certain individuals to compensate for neural decline in the aging brain. More specific, the cognitive reserve theory postulates that the differential recruitment of typical brain networks or the additional recruitment of other, compensatory, networks gives rise to the variability in task performance in the elderly (Stern, 2009; Steffener and Stern, 2012). Park and Reuter-Lorenz (2009) also elaborate on the efficient recruitment of additional neural networks, which they call scaffolding networks or scaffolds (Park and Reuter-Lorenz, 2009). In their scaffolding theory of aging and cognition (STAC), they argue that older adults showing high levels of cognitive functioning make effective use of scaffolding networks to maintain task performance. However, the link between life factors such as education and IQ on one hand and cognitive performance on the other should be interpreted with caution: some longitudinal studies have failed to find a reliable association between higher education and stability of cognitive performance with increasing age (Christensen et al., 2001; Ritchie et al., 2013). Furthermore, lower levels of education attainment and crystallized intelligence (or lower cognitive reserve) are not necessarily associated with general lower cognitive performance in the elderly; our results show that older age seems to be related to selective changes in particular cognitive domains. Which specific cognitive domains are affected seems to vary across individuals. While our results are in line with previous studies, it must be noted that the majority of our older participants had higher educational attainment (category 3 and 4; see Table 2). This skewed distribution in terms of education attainment may be considered a limitation of the current study. This limitation is unfortunately inevitable due to the voluntary nature of participation in this kind of studies.

The cognitive profile of two “older” subgroups may be of interest to clinical-decision making. An intriguing question is whether the cognitive profile observed in the poorest performing older subgroup (S5) is just a facet of healthy aging. An alternative explanation may be that decline in cognitive function exceeds the respite offered by compensatory strategies in participants in this group and that they are at a higher risk for developing mild cognitive impairment (MCI) or dementia. It should be noted however, that ours is not a longitudinal study so that we have no information on prior cognitive performance in any of our participants. One of the criteria for an MCI diagnosis is objective memory impairment for age, which is often conceptualized as performing 1.5 SD below the performance of age-mates (Petersen, 2004). Although participants in our subgroup 5 performed well below average on the included neuropsychological tests, they did not perform particularly worse at the included measures of memory for which their *z*-scores (calculated with respect to the entire group of participants) were above −1.5. Their MMSE scores did not differ from those of the other older subgroups either. Another interesting “older” group is subgroup 6, the cognitive profile of which was characterized by lower cognitive performance on tests measuring delayed and immediate recall and above average performance in the remaining cognitive tests. Performance differences in memory functions have been considered a defining feature that can distinguish participants diagnosed with MCI and healthy control subjects. In a study by Petersen et al. (1999), participants with MCI showed lower performance on memory functions while retaining comparable performance in other cognitive functions to the healthy control participants. Participants classified in subgroup 6 thus seem to exhibit the characteristics of MCI patients in the Petersen et al. (1999) study, despite their optimal MMSE scores and performance scores exceeding −1.5 SD below the average of all participants. For participants classified in both these cognitive profiles (S5 and S6), additional (longitudinal) information is needed to examine transition stages from healthy aging to aging-related neurodegenerative disorders. To this end, measures of differences in underlying neural activity may help to better understand performance variability among these “older” subgroups (Park and Reuter-Lorenz, 2009; Saliasi et al., 2013; Geerligs et al., 2014).

Thus, the results of our community structure detection analysis confirm the findings of several previous studies, which all identified heterogeneous cognitive profiles in elderly. A constant finding is the presence of a group of elderly with a cognitive profile that is characterized by generally lower cognitive performance (Ylikoski et al., 1999; Gunstad et al., 2006; Foss et al., 2009; Costa et al., 2013). The pattern of cognitive performance characterizing the other identified groups of elderly differed between the studies. However, findings suggest that several elderly show average and above average performance on several cognitive tests (Ylikoski et al., 1999; Gunstad et al., 2006; Foss et al., 2009; Costa et al., 2013). Adding to these results, our findings clearly indicate that some elderly achieve cognitive performance levels comparable to those found in certain younger participants.

Variability in cognitive performance was also observed in the young population. In particular, the younger subgroups showed consistently high performance on all but the verbal fluency tasks. One of these profiles was further characterized by relative underperformance in semantic fluency while the other showed an additional decrease in working memory span. It is known that younger adults generally have lower vocabulary knowledge than older adults (Kavé and Yafé, 2014), which probably explains their reduced capacity to generate words based on their semantic or phonetic properties. As an interesting consequence of our approach to determine cognitive profiles in a mixed population of young and older adults, some younger participants were classified in “older” subgroups with poor cognitive performance (S4 and S5), indicating that efficient cognitive performance does not characterize all younger adults. Our results confirm the presence of cognitive performance variability not only among older, but also among younger adults. As such, conclusions based on averaged performance levels in both young and old groups may obscure existing variability in cognitive functioning in either age group.

## Conclusions

The results of this study confirm the prominent effects of aging on cognitive performance, as the majority of younger and older adults were classified in age-related subgroups. However, most older persons only showed moderate decline in domain-specific cognitive performance, while one group of older persons still performed at a similar cognitive level as younger adults. This finding provides more evidence that the notion of inevitable cognitive performance decline at older age is too simple. Aging is not a unitary process and age-related differences in cognitive performance—as our study illustrates—can become apparent in variable patterns across cognitive domains, possibly because neuronal decline varies across brain areas for individuals. Moreover, we found that high performance levels in older persons are associated with higher education levels and higher IQ scores. This “performance-cognitive reserve” association suggests that high performing older individuals may be able to adequately cope with age-related changes in the brain.

## Conflict of Interest Statement

The authors declare that the research was conducted in the absence of any commercial or financial relationships that could be construed as a potential conflict of interest.

## Footnotes

^1^http://www.csie.ntu.edu.tw/~cjlin/libsvm/
